# Correlation of Oxidative Stress Biomarkers with Activity of Pediatric Idiopathic Nephrotic Syndrome

**DOI:** 10.3390/biomedicines13081984

**Published:** 2025-08-15

**Authors:** Matjaž Kopač, Aleš Jerin, Ema Bohinc, Joško Osredkar

**Affiliations:** 1 Department of Nephrology, Division of Paediatrics, University Medical Centre Ljubljana, Bohoričeva 20, 1525 Ljubljana, Slovenia; 2 Faculty of Medicine, University of Ljubljana, Vrazov trg 2, 1000 Ljubljana, Slovenia; 3 Institute of Clinical Chemistry and Biochemistry, University Medical Centre Ljubljana, Zaloška cesta 2, 1525 Ljubljana, Slovenia; ales.jerin@kclj.si (A.J.); josko.osredkar@kclj.si (J.O.); 4 Faculty of Pharmacy, University of Ljubljana, Aškerčeva 7, 1000 Ljubljana, Slovenia

**Keywords:** oxidative stress biomarkers, idiopathic nephrotic syndrome, children, oxidative stress index

## Abstract

**Background/Objectives:** This study investigated the correlation of oxidative stress biomarkers with the activity of idiopathic nephrotic syndrome (INS) in Slovenian children. **Methods:** In this prospective study, sequential plasma and urine samples from 20 children with INS in different phases of disease activity were taken: at first disease presentation or relapse (before glucocorticoid (GC) treatment), at time of remission achievement, and after discontinuation of GC treatment. This study measured oxidative stress biomarkers, such as 8-hydroxy-2′-deoxyguanosine (8-OHdG), hexanoyl-lysine (HEL) adduct, dityrosine (DiY), and 15-isoprostane F2t, using competitive enzyme-linked immunosorbent assay (ELISA) and assessed oxidative status using the FRAS 5 analytical system, which enables rapid photometric measurement of both oxidative and antioxidant capacity from biological fluids. Two complementary tests were performed: the d-ROMs test (derivatives of reactive oxygen metabolites) and the PAT (plasma antioxidant test). The oxidative stress index (OSI) was calculated as the ratio between them. **Results:** Concentrations of isoprostanes in urine were statistically significantly lower in patients at first disease presentation or relapse compared to time of remission achievement. Values of PAT test in serum were significantly highest after GC treatment. Values of d-ROMs test in serum were significantly lower at time of remission achievement compared to first disease presentation or relapse. Values of 8-OHdG, HEL, DiY (in plasma and urine), isoprostanes, and OSI in plasma did not statistically significantly differ in various phases of disease activity. **Conclusions:** Isoprostanes in urine and PAT in serum could serve as potential biomarkers of oxidative stress and disease activity in children with INS.

## 1. Introduction

### 1.1. Etiology and Pathogenesis of Idiopathic Nephrotic Syndrome in Children

Idiopathic nephrotic syndrome (INS) in children is a common kidney disorder characterized by nephrotic range proteinuria (above 40 mg/hour/m^2^ or urine protein/creatinine ratio above 200 mg/mmol), hypoalbuminemia (serum albumin concentration below 30 g/L), peripheral edema, and hyperlipidemia. Remission is defined as negativization of proteinuria and improvement of clinical status. The exact cause remains unclear, but the pathogenesis of the condition is generally believed to involve increased filtration of macromolecules across the glomerular capillary wall. The glomerular filtration barrier consists of fenestrated endothelial cells, the glomerular basement membrane (GBM, consisting of type IV collagen, laminin, heparan sulfate proteoglycans, and other proteins), and podocytes (highly specialized epithelial cells). Foot processes of adjacent podocytes interdigitate with 30–40 nm gaps that are maintained by the slit diaphragm, a zipper-like pattern membrane [1].

The filtration of macromolecules, such as large anions (albumin), across the glomerular capillary wall is normally restricted by charge selectivity, created by the endothelial cells and the GBM that have a net negative charge due to polyanions. In minimal change disease (MCD), the most common cause of INS in children, there is a loss of anionic charge that is not accompanied by any structural damage to the glomerular filtration unit observed by light microscopy; however, podocyte foot process effacement is noticed on electron microscopy. The glomerular capillary wall is also size-selective, having functional pores of approximate radius of 40–45 A that are located throughout the GBM and across the GBM at the slit diaphragms between adjacent epithelial cell foot processes [1]. Several mechanisms can lead to a glomerular injury in INS. It is thought to be mediated by a T-cell-driven immune response, leading to the release of cytokines or other circulating factors that damage the podocytes and thus influence the integrity of the glomerular filtration barrier, resulting in leakage of proteins into the urine. Some genetic predisposition to INS in children has been observed, particularly mutations in genes encoding for podocyte proteins, such as NPHS1 (nephrin) and NPHS2 (podocin). These mutations can predispose the glomeruli to damage and proteinuria [1].

### 1.2. The Role of Oxidative Stress in Pediatric INS

Oxidative stress has been proposed to play a significant role in the pathophysiology of INS and other kidney diseases in children. It arises from an imbalance between reactive oxygen species (ROS) production and the body’s antioxidant defenses, leading to cellular and tissue damage. Some studies identified malondialdehyde (MDA) as the most consistent and reliable diagnostic biomarker, showing elevated levels during active disease phases and correlations with disease severity, relapse, and steroid resistance [2,3,4]. In addition, antioxidant enzyme activity, such as superoxide dismutase (SOD), glutathione (GSH), and total antioxidant capacity (TAC), is decreased in active disease stages, reflecting reduced antioxidant defenses [5]. Improvement in antioxidant status (e.g., SOD, GSH) is observed during remission, correlating with treatment response [6]. Oxidative stress and antioxidant imbalance show dynamic fluctuations between relapse, remission, and therapy [5,6]. Composite measures like thiol/disulfide homeostasis (DTDH) and total antioxidant status (TAS) provide additional insights into disease activity and recovery [5]. Elevated oxidative stress markers (e.g., MDA, advanced oxidized protein products (AOPP)) are also linked to inflammatory processes, suggesting oxidative stress–inflammation interplay in INS pathology [7]. Antioxidant therapies and dietary interventions remain investigational, with some studies showing potential for improving TAC and reducing oxidative damage [8]. In addition, a recent study on animal models revealed that lithium, used in the treatment of human mental disorders, while its excessive accumulation in the body can cause kidney injury and nephrotic syndrome, caused inflammatory damage and reduced antioxidant enzyme capacity of glutathione peroxidase, total SOD, TAC, and catalase, and increased production of ROS [9]. However, evidence is limited and inconsistent.

In summary, there is robust evidence for some important oxidative stress biomarkers in pediatric INS, with promising applications for disease monitoring and treatment response prediction, though further standardization and broader validation of emerging markers are needed. The aim of this prospective study was to assess the role of some newer oxidative stress biomarkers activity, such as dityrosine (DiY), 8-hydroxy-2′-deoxyguanosine (8-OHdG), hexanoyl-lysine adduct (HEL), isoprostanes, and oxidative stress index (OSI) in the diagnostic evaluation at different disease stages and in longitudinal follow-up of INS in Slovenian children.

This study was conceived as a pilot investigation, enrolling a limited but well-characterized cohort of Slovenian children with INS. By capturing repeated measures at distinct clinical phases—first disease presentation or relapse, remission, and post glucocorticoid (GC) treatment—the design allowed within-subject comparisons, reducing inter-individual confounding and enhancing biological signal detection despite a small sample size.

## 2. Materials and Methods

### 2.1. Study Design

In this prospective single-center study, with 18 months duration, sequential blood and urine samples from children with INS in different phases of disease activity were taken: at first disease presentation or relapse (before initiation of GC treatment), at the time of remission achievement with GC treatment, and in persistent remission after discontinuation of GC treatment (at least two months after remission achievement), whenever feasible.

All children with a diagnosis of INS, either at its first presentation or at relapse, who were evaluated at our clinical department during the study period (between March 2023 and September 2024) were included in the study. All children with other forms of nephrotic syndrome, such as genetic or secondary nephrotic syndrome (in the context of another glomerular or systemic disease, for example), where etiopathogenesis is different, were excluded.

A written informed consent was obtained prior to sample collections from patients’ parents and patients if they were above 14 years old. The study was approved by Slovenian National Committee for Medical Ethics, number 0120-501/2022/3.

Clinical baseline information for all included INS pediatric patients is presented in Table 1.

In addition, some patients did not appear at follow up visits or stayed on low-dose GC for prolonged period after remission in order to prevent relapses. For these reasons, third samples (post GC) were not taken and also not second sample in Pt 20. In patients 2, 10, and 13, relapses were noted just in laboratory values (initial stage of relapse) at regular follow-up visits (when third set of samples, after GC, was planned) and, therefore, samples at relapse were taken twice in these patients. In patients 15–19, samples were taken just in stable phase of the disease, when in prolonged remission, long after GC therapy, because they did not experience relapses in the study period.

### 2.2. Oxidative Stress Biomarker Determination

Competitive enzyme-linked immunosorbent assay (ELISA) kits were used to measure 8-OHdG, HEL, DiY, and 15-isoprostane F2t concentrations (Japan Institute for the Control of Aging, JaICA, Shizuoka, Japan; catalog numbers presented in Table 2), specifically selected for their high sensitivity and reliability. All samples were run in duplicate. Standard curves were generated with 4-parameter logistic regression, and all assays showed high linearity (R^2^ > 0.98). Quality controls provided by the manufacturer were included in every assay run.

The 8-OHdG ELISA is a competitive assay that utilizes a monoclonal antibody specific for 8-OHdG and allows sensitive detection of oxidative DNA damage. The HEL ELISA detects early lipid peroxidation products bound to proteins. DiY, a marker of protein oxidation, is measured using a highly specific monoclonal antibody. The isoprostane ELISA enables quantification of lipid peroxidation via 15-isoprostane F2t, a prostaglandin-like compound formed non-enzymatically by free radicals.

All assays include internal quality controls (QCs), and the intra- and inter-assay coefficients of variation (CVs) were within acceptable limits, confirming the reproducibility and accuracy of the assays. Standard curves were generated in each assay run, and concentrations were calculated from the absorbance values using four-parameter logistic regression. Table 2 presents overview of ELISA kits used for oxidative stress biomarkers.

### 2.3. Evaluation of Oxidative Status Using d-ROMs, PAT and OSI

This study assessed oxidative status using the FRAS 5 analytical system (H&D srl, Parma, Italy), which enables rapid photometric measurement of both oxidative and antioxidant capacity from biological fluids, most commonly plasma or serum. Two complementary tests were performed: the d-ROMs test and the PAT.

To obtain a comprehensive estimate of oxidative stress, the OSI was calculated as the ratio between d-ROMs and PAT values, multiplied by a constant to adjust units.

All analyses were performed according to the manufacturer’s instructions. Internal quality controls were used in each batch to ensure precision and reliability. The intra- and inter-assay CV for both tests were within acceptable ranges. Table 3 presents parameters for oxidative status evaluation.

This study used SPSS version 27.0 (IBM Corp., Armonk, NY, USA) in order to do the necessary calculations. Each biomarker analysis included internal quality control and standard curve fitting with R^2^ > 0.98.

This study compared patients in three groups: those at first disease presentation or relapse (before initiation of glucocorticoid (GC) treatment—group 1 or G1), at the time of remission achievement (G2), and those in persistent remission after discontinuation of GC treatment (G3).

### 2.4. Statistical Methods

Statistical analysis in this study was carried out using Microsoft Office 16’s Excel computer program, in heading Formulae, subheading Statistics. Where data followed a normal distribution, unpaired t-test was used for between-group comparisons. Two-tailed distribution was applied in analysis. In cases of non-normality, the Mann–Whitney U test was applied. However, results were very similar and not significantly different. Statistical significance of concentration differences of various substances of interest in plasma and urine samples was presented as p-value, with values less than 0.05 considered statistically significant.

## 3. Results

This study included 20 patients with INS, aged between 3 and 19 years, 17 boys and 3 girls. Thirteen of them had kidney biopsy and all of them had MCD. Four of them were new cases of INS, eleven had relapse and five of them were caught in stable remission only, after discontinuation of GC treatment. Two of the included children were receiving GC treatment (on alternate days), four of them vitamin D, and seven of them other immunosuppresive drugs (four of them mycophenolate-mofetil (MMF), one cyclosporine, and two tacrolimus) at relapse. The concentrations of studied oxidants and antioxidants (DiY, HEL, 8-OHdG, isoprostanes) in serum and urine samples of patients are presented in Table 4 and the values of studied functional oxidative indices (d-ROMs, PAT, OSI) in serum samples of patients in different phases of disease activity are presented in Table 5.

This study detected statistically significantly lower concentrations of isoprostanes in urine samples of our patients at their first disease presentation or relapse (G1) compared to remission achievement (G2). Values of PAT in patients after GC treatment (G3) were statistically significantly higher compared to other two phases of disease activity. Values of d-ROMs test at remission achievement (G2) were statistically significantly lower compared to those in G1. Statistically significant threshold was defined as p-value less than 0.05. The concentrations of other studied substances and OSI were not statistically significantly different in various phases of disease activity.

Total oxidant status, as shown by the d-ROM values, was considerably higher during the active phase (G1) and decreased during e (G2), suggesting a decrease in oxidative stress after disease control.

PAT-measured antioxidant capacity was comparatively steady during active disease and remission, but it dramatically rose after GC treatment (G3).

It is of note that the highest values of isoprostanes in serum as well as in urine were detected in persistent remission after GC treatment (G3), in both cases in a patient with the most difficult clinical course of the disease, requiring prolonged hospitalization, several infusions of human albumins with furosemide, and even transient acute hemodialysis due to associated acute kidney injury.

Table 4 and Table 5 present the results in more detail.

This study examined DiY, isoprostanes and HEL, and 8-OHdG as biomarkers of oxidative damage to proteins, lipids, and DNA, respectively. Functional redox biomarkers (d-ROM, PAT, and OSI) were added to these to give a thorough oxidative stress profile at every stage of the disease.

**Figure 1:**
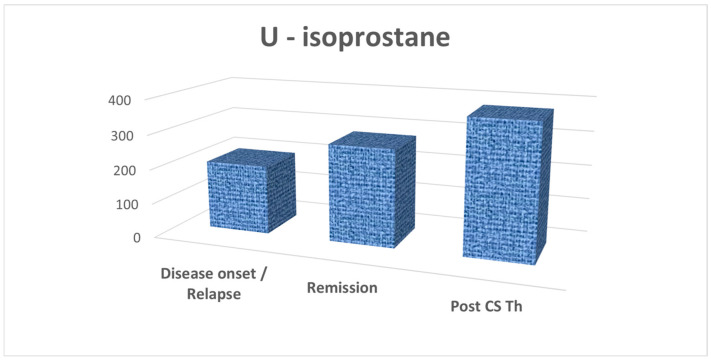
Average concentrations of isoprostanes in urine samples (U-isoprostane, in ng/mmol creatinine), with statistically significantly lower concentrations in patients at their first disease presentation (disease onset) or relapse, compared to those in remission, achieved by corticosteroid (CS) therapy (Th).

**Figure 2:**
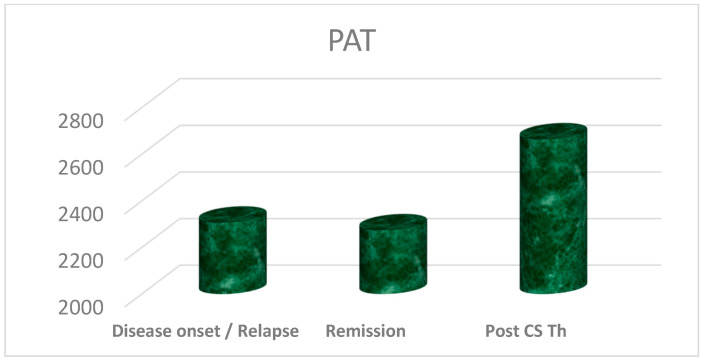
Average values of plasma antioxidant test (PAT, in µmol/L ascorbic acid) in serum samples of patients, with statistically significantly higher values in persistent remission, after corticosteroid (CS) therapy (Th), compared to other two phases of disease activity.

**Figure 3:**
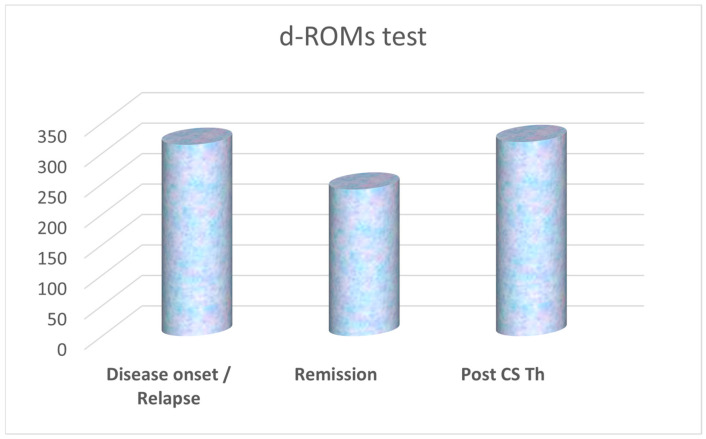
Average values of derivatives of reactive oxygen metabolites (d-ROMs test, in U.CARR units) in serum samples of patients, with statistically significantly lower values at remission achieved by corticosteroid (CS) therapy (Th), compared to those at first disease presentation (disease onset) or relapse.

**Figure 4:**
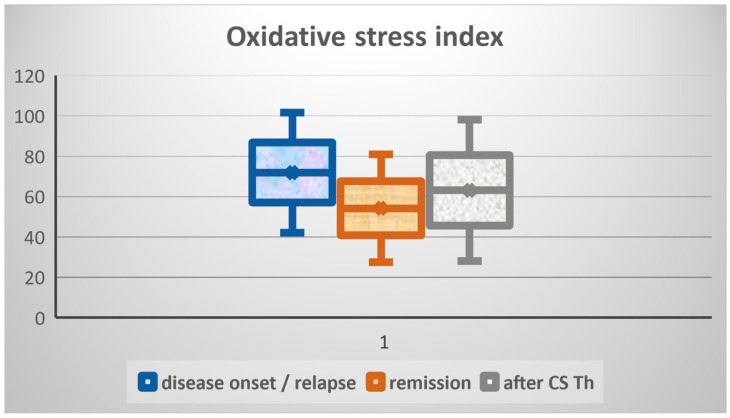
Average values of oxidative stress index (OSI, in arbitrary units) in serum samples of patients. The lowest values were detected at remission, achieved by corticosteroid (CS) therapy (Th), compared to other two phases of disease activity but with no statistically significant difference.

The results of average values of significantly different biomarkers, such as isoprostanes in urine, and PAT and d-ROMs test in serum, are presented in Figure 1, Figure 2, and Figure 3, respectively. Due to the small-to-moderate effect size and the variability of both d-ROMs and PAT, the OSI did not significantly change between illness stages, although being numerically highest during active disease. For this reason, OSI is presented as well, in Figure 4.

## 4. Discussion

### 4.1. Oxidative Stress Biomarkers in Kidney Disease

Derivatives of reactive oxygen metabolites (d-ROM), OSI, and the plasma antioxidant test (PAT) are examples of composite and dynamic oxidative stress markers that have received increased attention in recent years. These markers may offer further clinical value in determining the redox status in children with INS. By measuring hydroperoxides (ROOH), which are early-stage oxidative intermediates and indirect indicators of reactive oxygen species (ROS), the d-ROM test assesses the total oxidant capacity of plasma. Alkoxyl and peroxyl radicals are produced when these hydroperoxides react with transition metals, reflecting the body’s continuous oxidative activity [10]. The results are given in Carratelli units (U.CARR), where values above 300 U.CARR typically signal high oxidative stress [11].

In contrast, the PAT test measures the total plasma antioxidant capacity, which indicates the available antioxidant defense by evaluating biological fluids’ capability to convert ferric ions to ferrous ions (FRAP principle). PAT results are reported in U.CARR, as with d-ROM, and values greater than 2200 U.CARR are regarded as typical for systemic antioxidant capacity [12]. The ratio of d-ROM to PAT is multiplied by 100 to determine the OSI. Because of its sensitivity in detecting imbalances between oxidative damage and protective responses, this index offers a composite assessment of oxidative load in relation to antioxidant buffering capacity and has demonstrated encouraging potential in clinical settings [13]. Disproportionate oxidative stress is indicated by higher OSI values (>50), which are frequently linked to inflammatory and chronic disease activities [14].

A straightforward and repeatable point-of-care method for quantitative oxidative stress assessment in pediatric populations is provided by these parameters, which are measured by the FRAS5 analyzer. d-ROM, PAT, and OSI may represent early biochemical changes during relapse and remission in addition to current disease activity in nephrotic syndrome, where immunological dysregulation and inflammation are important factors. Although their importance in cardiovascular, metabolic, and autoimmune diseases has been demonstrated by earlier research, they are still few and mostly unexplored uses in juvenile nephrology [14,15]. A recent study on animal model explored the role and the underlying mechanism of Danggui Shaoyaosan (DSS) in nephrotic syndrome, induced by doxorubicin, after which inflammation and oxidative stress index were detected via ELISA. Proteinuria increased significantly; however, it was decreased by DSS therapy, together with blood urea and creatinine levels, in a concentration-dependent manner. According to these findings, DSS can protect against the development of nephrotic syndrome, mainly through improvement of podocyte injury and the inhibition of PI3K/Akt pathway-related proteins [16].

By comparing levels during active disease and remission phases and examining their correlation with therapeutic outcomes, this study seeks to examine the clinical value of d-ROM, PAT, and OSI in Slovenian children with INS.

F2-isoprostanes are regarded as one of the most reliable and accurate markers of lipid peroxidation in vivo among the many biomarkers of oxidative stress [17]. Both plasma and urine can include these prostaglandin-like substances, which are produced non-enzymatically by the peroxidation of arachidonic acid catalyzed by free radicals [18]. F2-isoprostanes are frequently used as reference markers in oxidative stress research and have been validated in a variety of clinical settings, including cardiovascular, kidney, and inflammatory diseases, because of their chemical stability, independence from cyclooxygenase pathways, and strong correlation with oxidative tissue injury [19,20]. F2-isoprostanes have encouraging potential as disease activity biomarkers in the setting of nephrotic syndrome, where oxidative lipid damage may be a contributing factor to podocyte dysfunction and glomerular injury.

F2-isoprostanes are regarded as stable and dependable byproducts of lipid peroxidation, whereas DiY represents protein oxidation and cross-linking caused by free radicals. HEL, on the other hand, is an early indicator of lipid peroxidation and 8-OHdG is a sensitive indicator of oxidative DNA damage. Furthermore, we used the PAT to assess total plasma antioxidant capacity and the d-ROMs test to measure circulating hydroperoxides (oxidant load). By combining the two metrics, the OSI was computed, offering a dynamic perspective on redox balance.

### 4.2. Implications of Oxidative Stress and Its Biomarkers (DiY, HEL, 8-OHdG, Isoprostanes, and OSI) in INS Children

Urinary 8-OHdG and related oxidative stress markers, particularly 8-oxoGuo and F2-isoprostanes, have robust evidence linking them to disease severity, steroid resistance, and oxidative burden in pediatric INS, while HEL and OSI remain significantly underexplored [21,22,23]. 8-OHdG and 8-oxoGuo have been strongly linked to oxidative DNA damage and mitochondrial dysfunction, with elevated urinary levels consistently correlated with worse INS outcomes, including steroid resistance [21,22]. A recent study revealed significantly higher creatinine and 8-OHdG in urine samples of urban compared to rural children while albumin, albumin to creatinine ratio (ACR), and thiobarbituric acid reactive substances (TBARS) were significantly higher in rural compared to urban children. In addition, TBARS was positively associated with creatinine and albumin in the cohort as well as in females and urban children, while 8-OHdG was positively associated with albumin in the cohort [24]. These markers have, therefore, shown potential as severity and progression indicators. In addition, F2-Isoprostanes reflect oxidative injury, lipid peroxidation, and reduced antioxidant potential, emphasizing ROS-induced damage in INS, with consistently higher levels observed in INS patients; they also provide stability as potential diagnostic markers [23]. However, only isoprostanes in urine (measured as isprostane/creatinine ration, expressed in µg/mmol creat.) have been detected with statistically significant difference in urine samples of our patients at their first disease presentation or relapse compared to remission. This substance could, therefore, serve as a biomarker of oxidative stress in our population of children with INS. On the other hand, HEL, a marker of lipid peroxidation, and OSI, touted as a global oxidative stress measure, have not been yet explicitly addressed, according to our knowledge, leaving their role unclear. They, together with other studied substances, have not been confirmed as reliable biomarkers of oxidative stress in our population of children with INS as well.

### 4.3. Oxidative Stress, Steroid Resistance, and Treatment Monitoring

Oxidative stress impacts responsiveness, with elevated oxidant markers (e.g., MDA, F2-isoprostanes) and reduced antioxidant defenses (e.g., serum TAC, sulfhydryl groups, and paraoxonase 1 (PON-1)) observed in steroid-resistant patients compared to steroid-sensitive groups [2,25,26]. PON1, an enzyme associated with high-density lipoprotein (HDL), has been confirmed to play a crucial role in hydrolyzing lipid peroxides, thereby protecting against oxidative damage. Reduced PON1 activity in NS patients may impair HDL’s antioxidant function, potentially leading to glomerular injury [26].

Bakr et al. connected oxidative stress to GC response in INS, proposing ROS as modulatory factors driving treatment dynamics [25]. The interplay between ROS and inflammatory pathways, such as pentraxin-3 and cytokines, is emerging as a key factor in steroid resistance, according to study by Mulat et al. where children with INS exhibited elevated oxidative stress markers during the acute phase of the disease, demonstrated by increased levels of AOPP and total oxidant status (TOS) during active disease phases. Conversely, antioxidant defenses such as PON1 activity were significantly reduced compared to remission phases. These findings suggest that oxidative stress contributes to the disease’s pathogenesis and progression [7]. However, our population of patients were all steroid-sensitive and, consequently, these biomarkers are of limited utility in this context. It is worth to mention that small cohorts and lack of longitudinal studies reduce the generalizability of these findings [27,28]. Systematic reviews exclude oxidative markers like 8-OHdG and HEL in favor of others like suPAR [27]. This could be, therefore, a challenge for future research direction, addressing these issues in more depth.

### 4.4. Biomarkers Specifically Linked to Oxidative Stress Severity in Pediatric INS

Urinary 8-OHdG levels were found to be elevated in pediatric INS, particularly during relapses. Kaneko et al. focused on 8-OHdG levels in children with INS, which emerged as one of the earliest markers linked to oxidative DNA damage and severity during relapses [21]. Later on, Liu et al. developed the concept of urinary oxidative stress biomarkers (such as 8-oxoGuo, 8-OHdG) as tools for stratifying disease severity, implicating ROS in mitochondrial and DNA damage [22].

Elevated TOS and AOPP are linked to severity in acute-phase INS and thus highlight oxidative stress as a driver of disease progression [7]. However, we decided not to evaluate these substances in our study. On the other hand, F2-isoprostanes have been found to be elevated in plasma and urine of nephrotic patients and could, therefore, serve as significant marker of lipid peroxidation and oxidative injury [23]. This has been partially confirmed in our study because isoprostanes in urine have been detected with statistically significant difference in urine samples of patients at their first disease presentation or relapse compared to samples of patients in remission.

8-OHdG has been proposed as a marker for poor prognosis and oxidative damage persistence in steroid-resistant cases [19]. Oxidative stress modulation has been proven to influence GC responses as increased stress is associated with resistance [25]. Our population of studied patients did not include steroid-resistant patients and, therefore, 8-OHdG and oxidative stress modulation cannot be applied as such a marker.

This study evaluated some other specific oxidative stress markers, such as DiY, which is formed through the oxidation of tyrosine residues in proteins and, therefore, serves as a marker for protein oxidation and has been associated with kidney damage [29,30]. In addition, this study evaluated HEL, an early marker of lipid peroxidation, reflecting oxidative modifications of lipids, which are prevalent in kidney diseases [29]. Despite the mechanistic relevance of both HEL and DiY as biomarkers of oxidative stress, our study did not identify statistically significant changes in their concentrations across different clinical phases of INS. Several potential explanations can account for this finding:

- Sample size limitations inherent to our pilot design likely reduced statistical power to detect subtle but biologically meaningful differences. The small cohort size, combined with inter-individual variability and heterogeneity in treatment timing, may have obscured real effects.
- The temporal dynamics of HEL and DiY may not align with the sampling points used in our study. HEL, as a marker of early lipid peroxidation, and DiY, reflecting protein oxidation through tyrosine cross-linking, may peak at different stages of oxidative injury than those captured (i.e., before overt clinical relapse or after inflammatory resolution). These markers might be more responsive to sustained oxidative insults than to transient fluctuations.
- Biological specificity may limit their utility in pediatric INS. For example, HEL has shown stronger associations with chronic oxidative conditions such as diabetic nephropathy or hypertensive kidney injury, where continuous lipid peroxidation plays a prominent role [31]. Similarly, DiY may better reflect cumulative oxidative protein damage in advanced or end-stage renal disease than in episodic relapses typical of steroid-sensitive INS [30].
- Finally, technical factors may have contributed to non-significant results. While all assays had acceptable intra- and inter-assay coefficients of variation, it is possible that sensitivity thresholds were insufficient to capture low-level oxidative changes in well-controlled patients.

Taken together, these findings suggest that although HEL and DiY are promising markers in other renal contexts, their utility in tracking short-term redox shifts in pediatric INS—especially in steroid-sensitive, early-phase patients—may be limited. Larger and stratified studies are needed to determine whether these markers have prognostic or diagnostic value in specific INS subtypes or chronic relapsing courses.

HEL and DiY can be, therefore, considered as emerging biomarkers for oxidative stress, with HEL indicating early lipid peroxidation and DiY reflecting protein oxidation through tyrosine cross-linking [32]. These markers have been studied in various contexts, including exercise-induced oxidative damage, diabetic complications, and atherosclerosis [32,33]. In pregnancy, elevated levels of oxidative stress markers, including HEL and DiY, have been associated with adverse outcomes. Specifically, high levels of propanoyl-lysine and 3-nitrotyrosine in maternal urine during the second trimester were linked to lower Apgar scores, while DiY showed a negative association with preterm birth risk [34]. These findings suggest that HEL and DiY may serve as valuable indicators of oxidative injury at different stages, potentially offering insights into various physiological and pathological processes.

Recent research highlights the critical role of oxidative stress in kidney diseases, including acute kidney injury (AKI), chronic kidney disease (CKD), and diabetic kidney disease (DKD). Oxidative stress, characterized by an imbalance favoring increased ROS generation, contributes significantly to kidney damage and associated cardiovascular risks [35]. While low ROS levels are essential for cellular functions, excess ROS can be pathological in CKD development [36]. Autophagy, triggered by oxidative stress, plays a protective role in kidney disorders by degrading intracellular oxidative substances and damaged organelles [37]. In DKD, redox regulation is complex, involving various reactive species with differing reactivities and specificities [38]. Another recent study about oxidative stress in the pathogenesis in endometriosis found no statistically significant differences in the erythrocyte levels of erythrocyte glutathione peroxidase (GPX), SOD, or the serum or peritoneal fluid levels of HEL and, therefore, these markers most likely do not contribute to endometriosis development [39]. Despite advances in understanding oxidative stress mechanisms in kidney diseases, clinical therapies targeting ROS and autophagy are still in early stages, highlighting the need for further research to develop effective treatments [37].

The statistically significant shift in U-isoprostanes (*p* = 0.0296) suggests a strong biological response, despite the relatively small absolute variations in U-isoprostanes and PAT between clinical phases. Due to their high chemical stability and susceptibility to oxidative alterations in vivo, F2-isoprostanes are well-established, trustworthy indicators of lipid peroxidation [18]. Even if the variations are not very noticeable, their directionality and statistical backing across several markers confirm their applicability. These results, which were acquired from a juvenile cohort that was carefully defined and subjected to internal control, provide credence to the possibility that these markers could serve as dynamic indicators of disease activity. However, we recognize that in order to improve threshold values, assess diagnostic sensitivity and specificity, and confirm their clinical application in everyday situations, larger and more varied cohorts are required.

Total oxidant status, as shown by the d-ROM values, was significantly higher during the active phase (G1) and decreased during remission (G2) in this study, suggesting a decrease in oxidative stress after disease control. The rise in G3 following treatment raises the possibility of an oxidative rebound following GC medication or, in certain cases, ongoing oxidative burden. The usefulness of d-ROMs in tracking disease activity and therapy response is supported by these patterns. The large range (min 53 to max 531 U.CARR) points to patients’ varied oxidative reactions.

PAT-measured antioxidant capacity was comparatively steady during active disease and remission, but it dramatically rose after GC treatment (G3), suggesting that the body’s antioxidant defense systems were strengthened, maybe as a result of GC anti-inflammatory and antioxidant properties. This may reflect a delayed compensatory antioxidant response or direct pharmacological effect of steroids on redox control.

Due to the relatively small effect size and the variability of both d-ROMs and PAT, the OSI did not significantly change between different disease stages, despite being numerically highest during active disease. Since OSI incorporates both oxidative damage and antioxidant buffering capability, its lack of statistical significance refers to either individual heterogeneity in redox homeostasis or inconsistent proportional variations in oxidative balance over timepoints.

We acknowledge that, under the constraints of our pilot study, the absolute differences in PAT and U-isoprostane levels between disease phases were statistically significant and biologically plausible, despite their small size. Systemic inflammation and redox imbalance can fluctuate within a limited physiological range in juvenile populations with INS [7], especially in early or well-controlled situations. For clinical decision-making, even slight but steady changes in oxidative stress markers can signal subclinical transitions like early relapse or incomplete remission. Furthermore, U-isoprostanes are regarded as the gold-standard indicators of lipid peroxidation, and numerous clinical settings have confirmed their sensitivity and repeatability. A temporal link with disease development is suggested by the considerable decrease in U-isoprostanes at the point of disease activity (*p* = 0.0296), followed by an increase during remission and after treatment. Likewise, the increase in PAT values following GC medication aligns with the established steroid-induced augmentation of systemic antioxidant capacity [40]. Trends in other redox markers, such as OSI and d-ROM, also support these findings, confirming the pattern shown.

The direction, internal consistency, and statistical support of the measured changes suggest a significant biological influence, despite their seemingly insignificant numerical appearance. The reliability of trend detection was improved because inter-individual variability was reduced due to the repeated sampling within the same patients in this investigation. In order to confirm clinical cut-offs and enhance diagnostic sensitivity and specificity, more research using bigger, stratified cohorts is required. However, when analyzed over time, our results imply that even minor biomarker changes could have significant clinical ramifications for disease surveillance in pediatric INS.

### 4.5. Limitations of the Study

We acknowledge the limitations of the study due to some inevitable confounding factors, including gender-specific effects on the observed outcomes, the influence of different medication regimens, and potential correlations with disease progression stages. However, subgroup analyses were constrained by sample size but are planned in future studies. In addition, despite certain immunosuppressive medications (such as tacrolimus or MMF) taken by some patients, these drugs were not able to prevent relapses in some of them, suggesting ongoing oxidative, immunological, or other pathological process despite of treatment. For this reason, we decided not to conduct additional analyses to evaluate potential influence of these drugs. Some basic data about use of these drugs in included patients are presented in Table 1. Clinical baseline information for all included INS pediatric patients. Regarding disease progression stages, all patients were quite homogenous in terms of typical presentation of INS, good response to GC treatment, and kidney biopsy findings (when indicated to be done).

The current study was set up as a pilot study to investigate the dynamics of redox biomarkers in a cohort of children with INS. Although within-subject comparison across disease phases was made possible by the repeated-measures methodology, we recognize that the overall sample size is still small and that the results should be regarded with caution. Furthermore, the data’s generalizability is restricted by the absence of stratification by gender or treatment mode. To validate the patterns seen and improve biomarker thresholds using bigger, multicenter cohorts, more research is required. However, the biological plausibility and therapeutic relevance of oxidative stress markers in this context are supported by the patterns’ consistency across several biomarkers and statistically significant alterations in a few chosen parameters. In addition, the repeated-measures design across distinct clinical phases allowed meaningful intra-individual comparisons, providing internally controlled evidence of oxidative stress biomarker dynamics.

These findings provide preliminary but promising evidence for the role of oxidative stress biomarkers—particularly d-ROM, PAT, and OSI—in the clinical assessment of children with INS. However, we recognize the need for further multicenter validation in larger, more diverse pediatric cohorts to establish reference values and evaluate predictive accuracy in routine practice.

## 5. Conclusions

Concentrations of isoprostanes in urine have been statistically significantly lower in patients at first disease presentation or relapse compared to those at time of remission achievement (*p*-value 0.0296). Value of PAT-measured antioxidant capacity, on the other hand, was comparatively steady during active disease and remission, but it dramatically increased after GC therapy (*p*-value 0.0109), suggesting strengthened body’s antioxidant defense systems, perhaps due to GC’s anti-inflammatory and antioxidant properties, reflecting a delayed compensatory antioxidant response or direct pharmacological effect of GC on redox control. Value of d-ROMs test was significantly decreased at time of remission achievement compared to the active phase, at first disease presentation or relapse (*p*-value 0.0458), suggesting a decrease in oxidative stress after disease control. Its increase in post-treatment period raises the possibility of an oxidative rebound following GC medication or, in certain cases, ongoing oxidative burden. The usefulness of d-ROMs in tracking disease activity and therapy response is, therefore, supported by these patterns. Due to the relatively small effect size and the variability of both d-ROMs and PAT, the OSI did not significantly change between different phases of disease activity, due to either individual heterogeneity in redox homeostasis or inconsistent proportional variations in oxidative balance over time. Isoprostanes in urine as well as PAT and d-ROMs test in serum could serve, therefore, as potential biomarkers of oxidative stress in children with INS. Monitoring oxidative stress biomarkers can aid in evaluating disease activity, progression, and response to therapy in pediatric kidney diseases, including INS. Markers like urinary 8-OHdG, 8-oxoGuo, and F2-isoprostanes provide consistent evidence for their role in assessing severity and prognosis in pediatric INS, only partially confirmed in our study. However, HEL and OSI require further investigation to determine their relevance. Further studies are warranted to validate these findings in larger populations and to define clinically actionable thresholds for routine use of these markers in pediatric nephrotic syndrome.

## Figures and Tables

**Table 1:** Clinical baseline information for all included INS pediatric patients.

Patient No.	Phase of Disease Activity	Proteinuria: 24 h Urine (g)/Spot Urine -prot./creat. Ratio (g/mol)	S-Albumin Conc. (g/L)	GC Dose (mg/kg/day)	GC Th Duration	Other Immunosuppressive Th
Pt 1	1st DP	ND/3106	15	0	0	no
	Remission	0.37/0	29	1	19 days	no
	Post GC	0/21	44	0	6 w. after GC Th	no
Pt 2	3rd relapse	0.76/42	36	1	3 days	no
	Remission	ND/0	41	1	7 days	no
	4th relapse	7.1/582	30	0	0	no
	Remission	0/0	40	1	10 days	no
	6th relapse	4.86/428	26	0	0	no
Pt 3	Relapse	5.55/ND	27	1	3 days	no
	Remission	0/13	35	1	7 days	no
Pt 4	1st DP	4.79/375	24	1	2 days	no
	Remission	0.16/0	30	1	15 days	no
	Post GC	0/ND	35	0	3 w. after GC Th	MMF (SDNS)
Pt 5	Relapse	5.53/ND	38	0	0	tacrolimus
	Remission	0/9	37	1	21 days	no
Pt 6	Relapse	ND/899	37	0.6 / 48 h (tapering)	28 w. (not full dose all the time)	MMF (SDNS)—stopped
	Remission	ND/17	33	1	20 days	tacrolimus
	Post GC	ND/20	44	0	4 w. after GC Th	tacrolimus
Pt 7	Relapse	1.15/ND	30	0	0	tacrolimus
	Remission	0/0	42	1	10 days	Tacrolimus
	Post GC	0/0	46	0	1 w. after GC Th	tacrolimus
Pt 8	1st DP	ND/1275	15	0	0	no
	Remission	0.22/ND	38	1	20 days	no
	Post GC	0/ND	40	0	3 w. after GC Th	Tacrolimus (SDNS, difficult clinical course)
Pt 9	Relapse	ND/1062	21	1	5 days	Cyclosporine
	Remission	ND/0	25	1	11 days	Cyclosporine
Pt 10	19th relapse	ND/510	48	0	0	no
	Remission	0/0	47	1	10 days	no
	21st relapse	0.5/174	47	0	0	no
Pt 11	Relapse	3.79/ND	29	0	0	no
	Remission	0.59/0	32	1	6 days	no
	Post GC	0.08/19	43	0	14 w. after GC Th	no
Pt 12	Relapse	9.66/ND	28	0	0	no
	Remission	0/0	43	1	11 days	no
Pt 13	Relapse	0.87/293	23	0	0	MMF (SDNS)
	Remission	0.41/21	28	1	7 days	MMF
	Relapse	0.56/73	35	0	0	MMF
	Post GC	0.08/0	42	0	17 w. after GC Th	MMF
Pt 14	1st DP	6.25/8419	30	1	1 day	no
	Remission	0/0	42	1	15 days	no
	Post GC	0.05/0	47	0	3 w. after GC Th	no
Pt 15	Post GC	ND/7	49	0	0	no
Pt 16	Post GC	ND/0	50	0	0	no
Pt 17	Post GC	0.14/11	45	0	0	MMF (FRNS)
Pt 18	Post GC	ND/7	50	0	0	no
Pt 19	Post GC	ND/0	41	0	0	no
Pt 20	Relapse	3.85/547	34	0	0	no

Legend: 1st DP—first disease presentation; Post GC—post glucocorticoid therapy; Th—therapy; GC—glucocorticoid; ND—not determined/no data; MMF—mycophenolate mofetil; w.—weeks; SDNS: steroid-dependent nephrotic syndrome; FRNS—frequently relapsing nephrotic syndrome; Proteinuria: values expressed as proteinuria in 24 h urine collection (g/24 h) and/or proteinuria in spot urine, expressed as protein/creatinine ratio (g/mol); nephrotic range proteinuria in 24 h urine collection is above approximately 1 g/24 h (in smaller children may be above cca. 500 mg) and protein/creatinine ratio above 200 g/mol in spot urine. Normal proteinuria is below 0.3 g in 24 h urine collection and protein/creatinine ratio below 20 mg/mmol in spot urine. In some urine samples, proteinuria was below limits of laboratory detection and expressed as 0. Notes: as described in Methods, first blood and urine samples were taken at first disease presentation or relapse, before initiation of glucocorticoid (GC) treatment, whenever feasible. However, in patients No. 2, 3, 4, 6, 9, and 14 they were taken after one to five days (in patient No. 6 with SDNS after 28 w., during tapering, not full dose of GC all the time) after start of GC therapy, mostly due to technical reasons, such as inability to take these samples during the evening or weekend (when admitted in these times).

**Table 2:** Overview of ELISA kits used for oxidative stress biomarkers.

Biomarker	Cat. No.	Sample Type	Sensitivity	Intra-assay CV	Inter-assay CV	Standard Curve Range
8-OHdG (DNA oxidation)	KOGHS-040914E	Urine, serum, plasma	~0.125 ng/mL	<10%	<15%	0.125–10 ng/mL
HEL (lipid peroxidation)	KHL-700/E	Serum, plasma	~1.0 ng/mL	<10%	<10%	2.6–624 nmol/L
Dityrosine (protein oxidation)	KDT-010/E	Serum, plasma	~0.05 ng/mL	<10%	<15%	0.05–12 µmol/L
15-Isoprostane F2t (lipid peroxidation)	KIP-050	Urine, plasma	~2.7 pg/mL	<10%	<15%	0.05–100 ng/mL

Legend: CV—coefficients of variation; 8-OHdG—8-hydroxy-2′-deoxyguanosine; HEL—hexanoyl-lysine adduct; ELISA—enzyme-linked immunosorbent assay; Cat. No.—catalog number.

**Table 3:** Parameters for Oxidative Status Evaluation.

Test	Principle	Measured Parameter	Units	Analytical Range	Intra-Assay CV	Inter-Assay CV
d-ROMs	Hydroperoxide-mediated oxidation of chromogen	Reactive oxygen metabolites	U.CARR	50–500 U.CARR	<5%	<8%
PAT	Reduction of Fe^3+^ to Fe^2+^ and chromogen complexation	Total antioxidant power	µmol/L ascorbic acid	600–2800 µmol/L	<5%	<7%
OSI (calculated)	Ratio of d-ROMs to PAT	Oxidative stress index	Arbitrary Units	-	-	-

Legend: CV—coefficients of variation; d-ROMs test—derivatives of reactive oxygen metabolites; PAT—plasma antioxidant test; OSI—oxidative stress index.

**Table 4:** The concentrations of studied oxidative stress biomarkers (dityrosine, HEL, 8-OHdG, isoprostanes) in serum (S) and urine (U) samples of patients in different phases of disease activity. The numbers of all samples are presented in a separate column while the numbers of samples in each group are not specifically presented, however, each group contained one third of all samples. Significant *p*-values are highlighted using bold text.

Substance (Units)	No.	First Disease Presentation/Relapse (G1)	Remission (G2)	Post GC Treatment (G3)	Max. Value	Min. Value	*p*-Value
S-dityrosine (µmol/L)	47	4.56 (1.69)	5.22 (2.17)	4.37 (2.76)	12.77 (G3)	1.24 (G3)	>0.05 (NS)
U-dityrosine (µmol/mmol creatinine)	47	0.22 (0.27)	0.29 (0.26)	0.17 (0.088)	1.09 (G1)	0.00 (G3)	>0.05 (NS)
S-HEL (nmol/L)	47	119.44 (88.31)	164.83 (292.39)	152.66 (117.86)	1202.44 (G2)	29.76 (G2)	>0.05 (NS)
U-HEL (nmol/mmol creatinine)	47	12.22 (5.59)	10.39 (6.10)	11.68 (9.46)	38.11 (G3)	1.33 (G3)	>0.05 (NS)
S-8-OHdG (µg/L)	42	5.72 (2.21)	6.43 (3.99)	7.47 (3.70)	20.00 (G2)	2.98 (G3)	>0.05 (NS)
U-8-OHdG (µg/mmol creatinine)	33	0.90 (0.82)	1.24 (0.51)	1.64 (0.90)	2.91 (G2)	0.05 (G1)	>0.05 (NS)
S-isoprostane (ng/L)	36	904 (1328)	1218 (2438)	1604 (3591)	9722 (G3)	8 (G2)	>0.05 (NS)
**U-isoprostane** (ng/mmol creatinine)	35	**200** **(100)**	281 (85)	383 (361)	1120 (G3)	44 (G3)	**0.0296** (G1 vs. G2)

Legend: Group G1: first disease presentation or relapse, before initiation of GC treatment; G2: at the time of remission achievement; G3: in persistent remission after discontinuation of GC treatment. Results are presented as average (standard deviation). The statistical significance of differences is presented as *p*-value. Abbreviations: No.—number of samples; G—group; NS—non-significant; S—serum; U—urine.

**Table 5:** The values of studied functional oxidative indices (d-ROMs, PAT, OSI) in serum sample of patients in different phases of disease activity. The numbers of all samples are presented in a separate column while the numbers of samples in each group are not specifically presented, however, each group contained one third of all samples. Significant *p*-values are highlighted using bold text.

Substance (Units)	No.	First Disease Presentation/ Relapse (G1)	Remission (G2)	Post GC Treatment (G3)	Max. Value	Min. Value	*p*-Value
d-ROMs test (U.CARR)	47	314.105 (115.60)	**240.667** **(90.47)**	318.692 (108.18)	531 (G1)	53 (G2)	**0.0458** (G1 vs. G2)
PAT test (µmol/L ascorbic acid)	47	2306.895 (413.02)	2274.067 (273.74)	**2664.077** **(431.02)**	3142 (G1)	1536 (G3)	**0.0274** (G1 vs. G3); **0.0109** (G2 vs. G3)
OSI (Arbitrary Units)	47	71.789 (29.85)	54.133 (26.78)	63 (35.08)	147 (G1)	21 (G1)	>0.05 (NS)

Legend: Group G1: first disease presentation or relapse, before initiation of GC treatment; G2: at the time of remission achievement; G3: in persistent remission after discontinuation of GC treatment. Results are presented as average (standard deviation). The statistical significance of differences is presented as *p*-value. Abbreviations: No.—number of samples; OSI—oxidative stress index; G—group; NS—non-significant.

## Data Availability

The data that support the findings of this study are available from the study’s principal investigator, M.K., upon reasonable request.

## Abbreviations

The following abbreviations are used in this manuscript:

ROS	reactive oxygen species
MDA	malondialdehyde
SOD	superoxide dismutase
GSH	glutathione
TAC	total antioxidant capacity
TAS	total antioxidant status
AOPP	advanced oxidized protein products
DiY	dityrosine
GC	glucocorticoid
CV	coefficients of variation
8-OHdG	8-hydroxy-2′-deoxyguanosine
HEL	hexanoyl-lysine adduct
ELISA	enzyme-linked immunosorbent assay
d-ROMs test	derivatives of reactive oxygen metabolites
PAT test	plasma antioxidant test
OSI	oxidative stress index
